# Frequency and Causes of Complaints against Emergency Medicine Specialists in Forensic Medicine Files; a Cross-Sectional Study

**Published:** 2019-01-27

**Authors:** Hossein Alimohammadi, Hamidreza Hatamabadi, Azita Khodayari, Mahmood Doukhtehchi Zadeh Azimi

**Affiliations:** 1 Emergency Department, Imam Hossein Hospital, School of Medicine, Shahid Beheshti University of Medical Sciences, Tehran, Iran.; 2 office of Tehran province forensic medicine commissions, Tehran, Iran.

**Keywords:** Emergency medicine, medical errors, malpractice, forensic medicine

## Abstract

**Introduction:**

Complaints against physicians have increased in recent years and one of the specialties facing a relatively high rate of complaints is emergency medicine. Therefore, the present study was designed with the aim of evaluating the frequency and causes of complaints against emergency medicine specialists in forensic medicine cases.

**Methods:**

In the present cross-sectional study, all the existing files in two forensic medicine centers, Tehran, Iran, from 2012 to 2015, in which complaints were filed against emergency medicine specialists, either alone or along with other physicians, were evaluated via census sampling method and their required data were extracted and recorded via a pre-designed checklist.

**Results:**

151 cases of medical complaints were filed against emergency medicine specialists during the study period. 85 (53.6%) complaints were filed following death of the patients and 66 (43.7%) were filed following an injury or disability. Multiple trauma, stomach ache, and altered level of consciousness were the most common chief complaints among young and old patients upon their ED visit. In 104 (68.9%) cases, the emergency medicine specialists were finally proved innocent. No significant correlation was found between the probability of proving innocent and the physician’s experience (p = 0.92), physician’s sex (p = 0.27), age range of the patient (p = 0.193), or the shift in which the patient had visited the ED (p = 0.32). The rate of proving innocent was significantly higher in complaints against governmental hospitals compared to non-governmental ones (73.6% vs. 61.9%; p= 0.004) and teaching hospitals compared to non-teaching ones (75.8% vs. 54.9%; p = 0.26).

**Conclusion:**

In about 70% of medical complaint cases against emergency medicine specialists, the in charge physician was proved innocent. No significant correlation was found between the probability of proving innocent and physician’s experience, the physician’s sex, the patient’s age range, or the shift in which the patient had presented to the ED.

## Introduction:

Emergency medicine is one of the young branches of specialty in medicine in which specialists are at a high risk of committing medical errors and malpractice and facing probable complaints due to it. More than 75% of emergency medicine specialists encounter these kinds of complaints at some point in their career (1). Studies have shown that emergency department (ED) is one of the most vulnerable departments to medical errors (2). ED is a special unit in which numerous and complex factors are at play, which make it potentially error prone (3). Emergency medicine specialty is in the third place regarding frequency of complaints in forensic medicine after obstetrics and gynecology, and orthopedics (4). Adverse outcomes caused by malpractice strongly correlate with complaints and financial penalties (5, 6).

For reducing the risk, 9 out of every 10 physicians use excessive drug precautions and diagnostic methods known as defensive medicine, which leads to an annual cost of 46 million dollars in the United States (7, 8). Fear of complaint can cause stress, depression, and reduction in the ability of effective communication with the patient in the individual providing service (9, 10). Of course, in many complaints the issue has been unavoidable or affected by factors out of the physician’s control (9).

Various methods exist for assessing patient safety such as hospital committees, periodical meetings of risk management, autopsy reports and reviewing the complaints filed in legal authorities (11).

Being aware of the number and types of complaints filed against emergency physicians as well as the evaluations performed by the board of judges can be of help in planning for preventing and reducing errors. Therefore, the present study has been designed with the aim of evaluating the frequency and causes of complaints against emergency medicine specialists in forensic medicine files.

## Methods:

***Study design and setting***

The present study is a cross-sectional study performed on the complaints filed against active emergency medicine specialists in Tehran province in 2 centers of western Tehran forensic medicine center and office of Tehran province forensic medicine commissions, Tehran, Iran, from 2012 to 2015. Protocol of the present study was approved by ethics committee of Shahid Beheshti University of Medical Sciences and researchers adhered to confidentiality of the evaluated files.

***Participants***

All the files related to complaints against emergency medicine specialists in forensic medicine centers of Tehran province were evaluated via census method. No limitation was considered regarding age, sex or job experience in the present study. In these files, the emergency medicine specialist was either one of the individuals, or the only person questioned regarding the probability of medical error or malpractice.

***Data gathering***

By referring to the archives of 2 centers, western Tehran forensic medicine center and office of Tehran province forensic medicine commissions, all the cases in which complaints were filed against emergency medicine specialists alone or along with other physicians were extracted. Then, using a pre-designed checklist, the required data including age and sex of the patient, chief complaint of the patient, cause of complaint against physicians, the result of evaluations performed by the board of forensic medicine judges, age and sex and job experience of the emergency medicine specialist and type of hospital (teaching or non-teaching) were extracted and recorded. The person in charge of gathering data was a senior emergency medicine resident under the supervision of an emergency medicine specialist.

***Statistical analysis***

Demographic characteristics were evaluated via descriptive statistics tests (frequency, mean and …). Chi-square and student’s t-test statistical analyses were used for performing comparisons and P-values less than 0.05 were considered significant. All the analyses were done using SPSS software version 18.

## Results:

**Figure 1:**
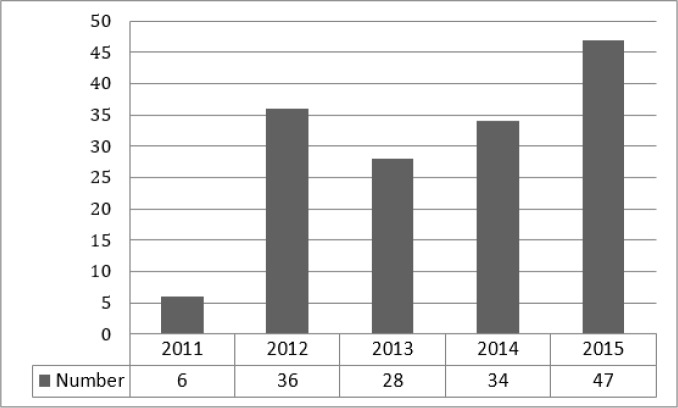
The frequency of medical complaints filed against emergency medicine specialists based on the studied years

151 cases of medical complaints were filed against emergency medicine specialists during the study period (39.7% against an emergency medicine specialist alone). Mean age of the patients in the mentioned files was 47.13 ± 21.62 (2-95) years (63.6% male). Table 1 has summarized the characteristics of the studied files. 85 (53.6%) complaints were filed following death of the patients and 66 (43.7%) were filed following an injury or disability. 121 (80.1%) cases were filed in governmental hospitals and 99 (56.5%) in teaching hospitals. Figure 1 has depicted the frequency of complaints based on the studied years.

***Outcome of the evaluated files***

In 104 (68.9%) cases, the emergency medicine specialists were finally proved innocent. The highest and lowest penalties considered for the physicians involved in the files were 5% and 45% of maximum penalty for murder. No significant correlation was found between the probability of proving innocent and the physician’s experience (p = 0.92), physician’s sex (p = 0.27), age range of the patient (p = 0.193), or the shift in which the patient had visited the ED (p = 0.32). The rate of proving innocent was significantly higher in complaints against governmental hospitals compared to non-governmental ones (73.6% vs. 61.9%; p= 0.004) and teaching hospitals compared to non-teaching ones (75.8% vs. 54.9%; p = 0.26).

## Discussion:

Based on the findings of the present study, in about 70% of medical complaint cases against emergency medicine specialists, the in charge physician was proved innocent. No significant correlation was found between the probability of proving innocent and physician’s experience, the physician’s sex, the patient’s age range, or the shift in which the patient had presented to the ED. Cases of governmental and teaching hospitals had a significantly higher percentage of proving innocent compared to others.

Studies have shown that currently we are faced with medical error crisis all over the world; as the number of complaints filed against the healthcare providing staff of hospitals is increasing in the United States and Europe, which is in turn associated with an increase in financial loss due to penalties (12-16). 

In this study, most complaints were filed against governmental and teaching hospitals. One of the reasons for complaints against emergency physicians working in governmental and university hospitals being more frequent can be that currently most emergency medicine specialists are working in these types of hospitals and their activities in non-governmental hospitals is much less than governmental hospitals. Thus, it is natural for the rate of complaints against governmental hospitals to be higher than non-governmental hospitals. However, despite the higher rate of complaints against these hospitals, the rate of proving innocent was significantly higher in governmental and teaching hospitals.

**Table 1:** Characteristics of the evaluated files in the present study

**Variable**	**Frequency (%)**
**Sex **	
Male	96 (63.6)
Female	55 (36.4)
**Age **	
< 18	8 (5.3)
18 – 60	92 (60.9)
> 60	51 (33.8)
**Chief complaint**	
Trauma	53 (35.1)
Stomach ache	26 (17.2)
Altered level of consciousness	15 (9.9)
General weakness	10 (6.6)
Chest pain	9 (6.0)
Shortness of breath	9 (6.0)
Other	29 (19.2)
**Time of presentation to ED**	
Morning sift (7 – 19)	89 (59.0)
Night shift (19 – 7)	62 (41.0)
**Complaint against **	
Emergency medicine alone	69 (39.7)
Multiple services	82 (84.3)
**Type of hospital**	
Governmental	121 (80.1)
Non-governmental	30 (19.9)
**Teaching hospital**	
Yes	99 (56.5)
No	52 (43.5)

ED: emergency department

In the present study, in about 70% of the complaints, the physician was proved innocent. In a study by Sadr et al. on orthopedics specialists also 61% of the cases led to the physicians proving innocent (17). This finding is in line with other studies performed on the subject of medical errors in Iran. In most of these studies, the physicians who were sued were proved innocent in the end in 60% to 80% of cases (18, 19).

In the present study, in 56% of the cases the reason for filing a complaint was death of the patient. This finding was also observed in the study by Gupta et al. (20), in which complaints due to death of the patient had a significantly higher rate compared to other complaints.

Multiple trauma, stomach ache, and altered level of consciousness were the most common chief complaints among young and old patients upon their ED visit. However, other complaints such as chest pain, shortness of breath and weakness were also very common and require more attention from emergency physicians at the time of examining patients. Alongside these cases, patients with laceration should be pointed out. Patients with laceration who visit the ED for its repair are among the most common patients in the ED, which have also recorded one of the highest rates of filing complaints against physicians, and physicians in ED need to apply scientific points and practical skills with more accuracy when encountering them and document the measures taken and explanations done to the patient with more care. In the study by Hwang et al. it was revealed that the most common diagnoses leading to complaints were infectious diseases, malignancies, and leg fractures. The study has analyzed complaints on medical errors in Taiwan for 12 years. The differences in the results can be due to the results of the current study being limited to the emergency department and different cultures of the 2 countries (4).

Another chief complaint that should be considered is loss of consciousness in elderly patients. Patients over the age of 65 years will be affected with altered level of consciousness with any small problem, which might be mistaken with amnesia or emotional disturbances in the elderly and finally lead to death or serious damage to the patient. Therefore, it is necessary that physicians in the ED evaluate these patients with more precision. Misdiagnosing the disease is one of the most common, most costly and the most dangerous medical errors (21). In a recent report, complaint due to misdiagnosis was on the top of the list of complaints related with malpractice in the United States (16). 

Finally, it should be noted that training emergency medicine specialists regarding cases leading to medical errors in order to prevent recurrence of similar errors, planning the shifts of emergency medicine specialist in a manner that they have enough down time for recovery, providing necessary equipment for ED, aiding in facilitation of patient turnover, employing enough nursing and service providing staff, and providing continuous education for increasing the scientific knowledge of emergency medicine specialists could be among the possible solutions for effectively reducing the prevalence and frequency of medical errors in ED.

Limitations

In the present study, only medical errors that have led to a complaint are evaluated, while at least part of them are never detected by the patients or their relatives or are not filed for complaints. Therefore, the results of the present study only reflect the part of medical errors that have led to a complaint. A more analytical evaluation of systematic errors that lead to medical errors has not been performed in the present study, which can be the subject of future studies.

## Conclusion:

Based on the findings of the present study, in about 70% of medical complaint cases against emergency medicine specialists, the in charge physician was proved innocent. No significant correlation was found between the probability of proving innocent and physician’s experience, the physician’s sex, the patient’s age range, or the shift in which the patient had presented to the ED. Cases of governmental and teaching hospitals had a significantly higher percentage of proving innocent compared to others.
